# Crohn’s & Colitis Foundation’s Advanced Practice Provider Preceptorship in Inflammatory Bowel Disease: Addressing the Knowledge Gap

**DOI:** 10.1093/crocol/otaf039

**Published:** 2025-06-10

**Authors:** Maureen Kelly, Abigail Meyers, Kate Carmody, Michele Rubin

**Affiliations:** University of North Carolina at Chapel Hill, USAmkelly@email.unc.edu; Medical College of Wisconsin, USA; Crohn’s & Colitis Foundation, USA; University of Chicago Medicine, USA

**Keywords:** Crohn’s & Colitis Foundation, inflammatory bowel disease, advanced practice providers, preceptorship

## Abstract

**Background:**

Inflammatory bowel disease (IBD) management has become increasingly complex and specialized education for advanced practice providers (APPs) is limited. The Crohn’s & Colitis Foundation’s Advanced Practice Provider Preceptorship was developed to educate IBD APPs to minimize this knowledge gap.

**Method:**

APP applicants were chosen based on their limited IBD knowledge and experience. Accepted applicants spent 3 days in an IBD center observing and learning. Pre- and post-surveys evaluated satisfaction, increase in knowledge, and confidence to manage IBD. A 3-month survey assessed mentorship and changes in practice.

**Results:**

Data measurement assessed satisfaction, knowledge, and confidence. The program grew from one participant in 2017 to 15 participants in 2023, with the maximum number of participants at 16 in 2021. From 2018 to 2023, knowledge and confidence from pre- to post-program improved. From 2018 to 2022, more than 75% of participants reported feeling “well-versed” to “extremely well-versed” in IBD knowledge after completion of the program. From 2019 to 2023, greater than 90% of participants reported feeling “moderately to very confident” or “completely confident” post-program.

**Conclusions:**

The Crohn’s & Colitis Foundation’s APP Preceptorship, a program for APPs with limited IBD knowledge and experience, is associated with program satisfaction and improved knowledge and confidence in treating IBD. Unexpected outcomes include changes in individual practices and ongoing mentorship. Continuation of this program will further enhance the IBD education of future APPs.

## Graphical Abstract

**Graphical Abstract:**
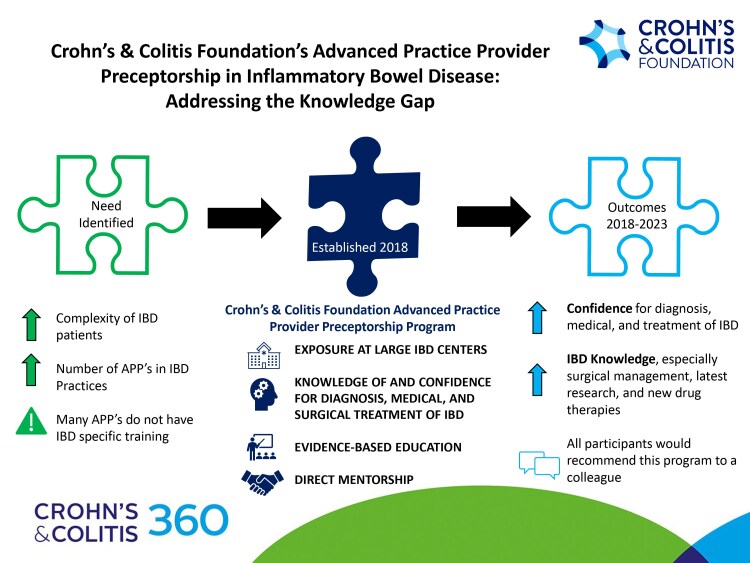

**Key Messages**

**What is already known?**

APPs are increasingly hired into practices to manage the care of IBD patients; however, specialized IBD training is limited particularly for APPs located in rural areas with less resource access.

**What is new here?**

Recognizing the gap in APP knowledge to care for IBD patients, the Crohn’s & Colitis Foundation’s National Scientific Advisory Nurse and Advanced Practice Provider Committee established the “APP IBD Preceptorship Program” to provide education to APPs with limited IBD knowledge and resources.

**How can this study help patient care?**

The Crohn’s & Colitis Foundation’s APP IBD Preceptorship Program aids in equipping the next generation of APPs with knowledge, confidence, and skillsets to provide quality IBD patient care.

## Introduction

Crohn’s disease (CD) and ulcerative colitis (UC), along with other subtypes, are forms of inflammatory bowel disease (IBD). Millions of Americans are living with IBD. Both CD and UC are characterized by periods of exacerbation and remission due to chronic inflammation of the gastrointestinal tract. Symptoms of IBD include but are not limited to, diarrhea, urgency, abdominal pain, bloody bowel movements, poor appetite, weight loss, and skin, eye, and bone problems. Over time, IBD can have a debilitating effect on quality of life, and it is imperative that patients are diagnosed early by experienced providers. IBD is costly, with patients experiencing a 3-fold higher annual cost for care than those without IBD.^1^ It is important to ensure access to high-quality care for these patients. Treatment primarily consists of a treat-to-target approach with medical therapy and surgical interventions. A multidisciplinary care team approach in IBD often includes gastroenterologists, surgeons, advanced practice providers (APPs), primary care providers, social workers, dietitians, and mental health providers.

As defined by the Centers for Medicare and Medicaid Services, APPs include nurse practitioners (NP), physician assistants (PA), certified nurse midwives (CNM), certified nurse anesthetists (CRNA), and clinical nurse specialists (CNS) who have advanced graduate level education and training, are board certified in their state and are independent providers who can offer a unique skill set for managing medical problems within their scope of training. They may work in hospital inpatient and/or outpatient ambulatory care settings providing medical care to IBD patients needing emergent or routine follow-up appointments to diagnose, treat, and monitor disease progression and remission. APPs play an important role in addressing barriers to communication between the patient and physician provider, assessing and referring patients with concerns regarding psychosocial health, nutrition, immunizations, and other preventive care.^2^ Most NP, PA, and CNS APPs do not receive specialized IBD education during their academic training and receive general gastroenterology education. Postgraduate NP/PA Fellowship programs are not required to enter the workforce as an NP/PA. There are limited postgraduate opportunities in the form of NP/PA Fellowships, and none particularly focusing on a subspecialty of gastroenterology, like IBD. APPs practicing in rural areas have less access to the resources needed to become experts in caring for patients with this chronic condition. In a study by Malter et al., APPs were considerably less comfortable than physicians in counseling regarding diagnostic testing, rendering an IBD diagnosis, evaluating phenotype and severity, treating perianal CD, proposing monotherapy vs. combination therapy, and surgery as a treatment of choice.^3^ Extra training in IBD is valuable as APPs expand access to care and improve patient and/or caregiver satisfaction, engagement, and education since they are often able to spend more time educating and counseling patients.^4^ However, specialized IBD training for APPs is limited. IBD-specific education is needed for APPs as IBD is a complex, challenging disease to treat and the field of IBD is constantly evolving with new medical and surgical treatments, as well as the addition of clinical practice recommendations and guidelines.

## Role of Crohn’s & Colitis Foundation’s Advanced Practice Provider Preceptorship in Inflammatory Bowel Disease Program

The Foundation’s National Scientific Advisory Committee (NSAC) Nurse and Advanced Practice Provider (RN/APP) Committee, which was formed in 2010, recognized the growing utilization of APPs in IBD and the need to minimize the APP knowledge gap for patients with IBD in both adult and pediatric care. The Foundation’s APP Preceptorship Program was initiated to support and provide education to APPs who had limited IBD knowledge, were unable to attend IBD-specific conferences, yet were practicing in the field of IBD.

### Program Goals:

- Gain exposure to complex clinical practices in leading IBD centers with the opportunity to observe members of the multidisciplinary team managing patient care.
- Increase knowledge of and confidence in using evidence-based practice approaches for the diagnosis, medical treatment, and surgical management of IBD.
- Apply evidence-based approaches to diagnosing, treating, and managing patients in clinical practice.
- Provide ongoing mentorship and networking opportunities to further increase their IBD knowledge and professional engagement.

### Funding

The program was initially unfunded and thus had one participant in 2017. The Foundation, in consultation with NSAC RN/APP Committee members, applied for and received grant funding from pharmaceutical companies, enabling 8 participants to rotate with travel expenses covered in a pilot program.

After the success of the 2018 program, the program expanded to 15 participants for the following annual cycle. Funding was expanded to include stipends to the participating IBD centers, for the time and expertise dedicated to leading the program at their respective sites. Finally, funding supported supplemental educational material for participants, marketing, and staff management fees.

### Administration

The Crohn’s & Colitis Foundation served as the central organization in executing the administrative and logistical components of the program. This included applying for funding, marketing the program, facilitating the application review process with the faculty workgroup, coordinating the placement of participants with host institutions, conducting pre- and post-evaluations, and reporting program evaluation results. The Foundation, NSAC RN/APP Committee faculty workgroup, and institution faculty directors closely collaborated to ensure a successful experience.

### Setting

The participating IBD centers initially included three adult sites: Massachusetts General Hospital, Mayo Clinic Rochester, and University of Chicago Medicine as well as a pediatric site at the University of North Carolina at Chapel Hill. From 2019 forward, the program expanded to include Cedars-Sinai as another adult site. An assigned APP program director at each facility coordinated the program, which included: identifying the faculty team, defining roles and responsibilities, and providing a detailed agenda of the preceptee’s daily activities aligning with program learning objectives.

### Core Curriculum

The adult and pediatric core curriculums (see appendix) reflected the 3-day preceptorship, which entailed preceptees observing expert IBD faculty managing patient care and included supplemental materials to address the learning objectives of increasing knowledge and confidence in diagnosis, treatment, and long-term management of patients with IBD. The curriculum included, yet was not limited to, materials highlighting diagnosis, new medical therapies, risks and benefits of treatment, treat-to-target strategies, nutrition optimization, psychosocial evaluation, surgical interventions, and related new and ongoing clinical research. Each center provided additional experiences, such as lectures, multidisciplinary patient conferences, endoscopy observation, in-patient rounds, and case studies on IBD. Preceptees completed learning checklists, which the program director signed off on at the completion of the three-day preceptorship, demonstrating objectives were met during their preceptorship.

### Measurable Outcomes

Preceptees evaluated their experience through pre-, post-, and three-month follow-up surveys, assessing: program satisfaction, knowledge of evidence-based approaches, and confidence in the ability to utilize evidence-based approaches for the diagnosis, treatment, and management of IBD.

- Program satisfaction through scales in various areas, including expectation and quality.
- Knowledge of evidence-based approaches to IBD diagnosis, management, and treatment of IBD.
- Confidence in the ability to utilize evidence-based approaches for the diagnosis, treatment, and management of IBD.
- Intention to apply the program learnings to their clinical practices.
- Perceived value of mentorship.

### Promotion

Marketing materials promoting the program’s open application were posted on the following websites: Crohn’s & Colitis Foundation; North American Society for Pediatric Gastroenterology, Hepatology and Nutrition (NASPGHAN); and Improve Care Now (ICN).

### Application and Selection Criteria

The applicants completed an online application, which asked the following:

- What prior experience has sparked your interest in inflammatory bowel diseases?
- What do you hope to gain from this program?
- What are your career plans?

Applicants submitted a curriculum vitae and a letter of recommendation from a collaborating physician. Crohn’s & Colitis Foundation staff completed objective scoring, and the faculty workgroup completed subjective scoring individually, which included rating open-ended responses and the letters of recommendation submitted by collaborating physicians. Initially, the program was open to all APPs; over time, participation was narrowed to those new to the field of IBD, to make the greatest impact in knowledge and confidence gains.

After scores were finalized, selected candidates were notified of their acceptance and placed at a host institution. HIPAA requirements and criminal background checks were completed by each preceptee prior to the program. Institution-specific requirements for participation were completed by each preceptee and faculty host site.

### In-Person Format

The program directors contacted each preceptee prior to the experience to gain an understanding of their individual specific needs and goals. When possible, this was incorporated into the program. After the needs assessment, the live program was planned over a three-day period. The in-person program has occurred annually since 2017; except when it occurred virtually during the 2020–2021 cycles due to the COVID-19 pandemic.

### Virtual Format

In 2021, the program was converted to an all-virtual program due to continued COVID-19 pandemic restrictions. The virtual program was held twice in 2021 for approximately the same total number of hours as the in-person format, but it was spread out over one week to try to avoid online fatigue. In addition, the University of Chicago Medicine faculty were able to perform virtual patient visits with preceptees during this time, focusing on patient and provider relationship building, engagement, shared decision-making, and education.

Regardless of in-person or virtual formats, preceptees were given take-home educational materials, including access to PowerPoint instructional IBD slides, IBD tip books, and Foundation resources online. All preceptees received a complimentary registration to the Crohn’s & Colitis Congress the following year.

## Results

Data measurements began in 2018 and included a quantitative pre-program assessment of knowledge and confidence, a quantitative post-program assessment of knowledge and confidence, and a qualitative three-month follow-up assessment illustrating continued communication between faculty director(s) and preceptee(s). The intention of the data measurement was to assess participant perception of knowledge, confidence, and overall satisfaction. With each new cycle, the data measurements varied to measure different data points. Data reflected are limited in some years.

**Figure 1:**
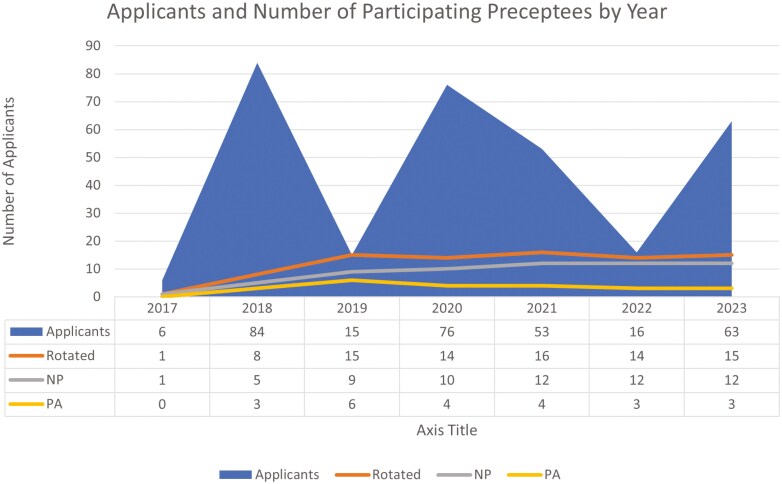
Number of preceptee applicants and participants per cycle, with APP credentials indicated. Data was collected via online applications.

Demographic information was obtained each year as represented in Figure 1. From 2017 to 2023, the number of applicants ranged from 6 to 84, with an average of 44.7 applicants. The number of participating preceptees ranged from 1 to 16, with an average of 11.8 preceptees. Of the total participants, 73% were nurse practitioners (NPs) and 28% were physician assistants (PAs).

Post-program surveys were distributed throughout all years. For each cycle, all participants reported that they, “would recommend this program to their colleagues.” In 2019, 2020, 2021, and 2023, all participants “planned to stay in contact with their Faculty Directors for ongoing mentorship.” In 2022, 86% of participants intended to “keep in contact with faculty for future mentorship.”

Participant perception of IBD knowledge was surveyed pre- and post-program in the following areas: diagnostic tools, indications for an interpretation of diagnostic testing, pathology, radiology, endoscopy, basic treatments, new drug therapies, surgery, complications, preventative care, nutrition, psychosocial issues, latest research, and special populations. Participants were asked to self-assess their knowledge in the following categories: no knowledge, limited knowledge, well-versed knowledge, and extremely well-versed knowledge. In all years of data collection, participants reported an increase in IBD knowledge from pre- to post-program (Table 1). The top three areas that consistently had a large knowledge gain, from well-versed to extremely well-versed post-program, were surgery, latest research, and new drug therapies.

**Table 1:** Preceptee self-reported ratings of “knowledge levels” across 14 topics, reflecting both pre-program and post-program self-assessments, 2018–2023.

	Well-versed to extremely well-versed									
	2018 *(8 respondents)*		2019 *(15 respondents)*		2020–2021 (Virtual) *(30 respondents)*		2022 *(14 respondents)*		2023 *(15 respondents)*	
**IBD knowledge**	**Pre-program**	**Post-program**	**Pre-program**	**Post-program**	**Pre-program**	**Post-program**	**Pre-program**	**Post-program**	**Pre-program**	**Post-program**
Diagnostic tools	100.00%	100.00%	86.67%	100.00%	N/A	N/A	64.29%	100.00%	53.33%	100.00%
Indications for and interpretation of diagnostic testing	75.00%	100.00%	80.00%	100.00%	N/A	N/A	50.00%	100.00%	60.00%	100.00%
Pathology	37.50%	100.00%	53.34%	100.00%	N/A	N/A	7.14%	78.57%	20.00%	73.34%
Radiology	25.00%	100.00%	53.34%	100.00%	36.00%	92.00%	14.29%	78.57%	33.33%	66.67%
Endoscopy	37.50%	87.50%	73.34%	100.00%	N/A	N/A	50.00%	92.85%	33.33%	93.33%
Basic treatments	75.00%	100.00%	86.67%	100.00%	N/A	N/A	64.29%	100.00%	60.00%	100.00%
New drug therapies	12.50%	100.00%	40.00%	100.00%	29.00%	86.00%	21.43%	100.00%	26.67%	86.66%
Surgery	0.00%	87.50%	20.00%	100.00%	7.00%	86.00%	22.06%	92.86%	0.00%	86.66%
Complications (obstructions, extra-intestinal manifestations)	50.00%	100.00%	66.67%	100.00%	N/A	N/A	50.00%	100.00%	26.67%	99.97%
Preventative care (vaccinations, screenings, smoking)	100.00%	100.00%	73.34%	100.00%	N/A	N/A	71.43%	100.00%	46.67%	100.00%
Nutrition	62.50%	100.00%	33.34%	100.00%	29.00%	93.00%	28.57%	92.86%	33.33%	80.00%
Psychosocial issues	62.50%	100.00%	60.00%	100.00%	43.00%	93.00%	28.57%	100.00%	33.33%	100.00%
Latest research	0.00%	75.00%	20.00%	86.66%	21.00%	93.00%	7.14%	92.86%	6.67%	40.00%
Special populations (pregnancy)	N/A	N/A	N/A	N/A	N/A	N/A	14.29%	92.85%	13.33%	26.66%

Data was obtained through online post-program survey form.

In 2018, the top 4 areas demonstrating the largest perceived knowledge improvement in the category of well-versed to extremely well-versed were surgery, new drug therapies, latest research tied with radiology, and pathology. In the pre-program survey, diagnostic tools (100%) and preventative care (100%) demonstrated the highest level of knowledge.

In 2019, the top 4 areas demonstrating the largest perceived knowledge improvement in the categories of well-versed to extremely well-versed were surgery, latest research tied with nutrition, new drug therapies, and pathology tied with radiology. The areas with the highest pre-program knowledge were diagnostic tools (86.67%) and basic treatments (86.67%).

In the 2020 and 2021 cycles, programming and participation were virtual, and data was combined. The top areas of post-program knowledge growth, described as feeling well-versed to extremely well-versed in descending order from what was measured, were surgery, latest research, nutrition, new drug therapies, radiology, and psychosocial issues.

In 2022, the top 5 areas demonstrating the largest perceived knowledge improvement in the category of well-versed to extremely well-versed were the latest research, new drug therapies, special populations, pathology, and psychosocial issues. The areas with the highest perceived IBD knowledge pre-program were preventative care (71.43%) and diagnostic tools (64.29%), which tied with basic treatments (64.29%). Two participants reported the post-program knowledge as “not applicable,” one each in the areas of pathology and radiology. Additionally, one participant did not answer the post-program knowledge question in the area of pathology.

In 2023, the top 5 areas demonstrating the largest perceived knowledge improvement in the category of well-versed to extremely well-versed were surgery, complications, psychosocial issues, endoscopy, and new drug therapies. The highest area of pre-program IBD knowledge was basic treatments tied with indications for an interpretation of diagnostic testing, followed closely by diagnostic tools (53.33%) and preventative care (46.67%).

Beginning in 2019, participants were asked to assess their perception of confidence in treating patients with IBD in the following areas: diagnosis, treatment, and long-term management. Participants reported an increase in IBD confidence from pre- to post-program (Table 2) for each year of data collection. In 2019, there was a higher level of pre-program IBD confidence than in subsequent years.

**Table 2.** Preceptee self-reported ratings of “confidence levels” in 3 topics: Diagnosis of IBD, treatment of IBD, and long-term management of IBD, 2019–2023

	Moderately confident or very confident or completely Confident							
	**2019** *(15 respondents)*		**2020–2021 (Virtual)** *(30 respondents)*		**2022** *(14 respondents)*		**2023** *(15 respondents)*	
	Pre-program	Post program	Pre-program	Post program	Pre-program	Post program	Pre-program	Post program
**Diagnosis of IBD**	80%	100%	21%	93%	42.85%	100%	53.34%	100%
**Treatment of IBD**	80%	93.33%	14%	93%	50.00%	100%	40.00%	100%
**Long-term management of IBD**	80%	100%	14%	93%	50.00%	100%	40.00%	100%

Data were obtained through online post-program survey form.

## Discussion

With the increase in IBD prevalence and the rising costs of healthcare, APPs are integral in providing high-quality healthcare to the IBD population; however, most APPs have had limited training in IBD. Advanced practice providers involved in the Crohn’s & Colitis Foundation NSAC RN/APP Committee recognized the gap in knowledge and created a preceptorship program with the purpose of educating APPs in management of the patients with IBD. The preceptorship has evolved and grown since the inaugural preceptee in 2017, now with 5 IBD centers and a total of 15 preceptees participating each year. The program continues to grow with a goal of hosting 19 preceptees in the 2024 cycle.

The program experienced the worldwide challenge of the COVID-19 pandemic and ultimately created an entirely virtual component to continue to enhance APP knowledge in IBD. The primary goal of increasing IBD knowledge was achieved while also focusing on keeping the program interactive despite the virtual platform. From the virtual experience grew new ideas and means of connections. Case-based presentations and hosting multiple preceptees at one time were benefits arising from the forced virtual programming. In addition, telehealth visits have continued in certain patient situations, allowing for better reach to IBD patients in rural communities. Having the preceptees present a case of their own at the end of the program proved to be beneficial, after hearing multiple faculty present cases and seeing more complex patients during clinic observations. Benefits beyond closing the knowledge gaps are recognized year after year.

Over time, the ongoing mentorship and communication between host sites and the preceptees are reported as a major benefit and satisfaction point for previous attendees, with some contact occurring beyond three years. Another unexpected benefit was the eagerness of preceptees to return to their institutions and share what they had learned and, in some cases, implement best practice changes. Preceptees focused on 2 or 3 areas that could be improved upon at their center, in addition to helping to establish more multidisciplinary care for IBD patients. There is professional growth beyond the program and their institutions with some previous preceptees becoming involved in local and national IBD organizations, presenting a poster or speaking, and, for 3 APP preceptees, applying for and being accepted into the NSAC RN/APP Committee, furthering their involvement in IBD.

## Conclusions

The Crohn’s & Colitis Foundation Advanced Practice Provider Preceptorship Program has been growing and evolving since its pilot in 2017. The benefit to attendees goes beyond bridging the knowledge gap for APPs on IBD. Additional benefits include networking and one-on-one mentorship. There is self-reported improved confidence in treating complex IBD patients and professional growth in local and national IBD organizations. Previous attendees promoted change within their own institutions to improve the care of IBD patients. The preceptorship program will continue to grow to promote the enhancement of high-quality APP-delivered IBD care to all patients.

As the information provided in this paper demonstrates, the Crohn’s & Colitis Foundation APP Preceptorship Program provides exceptional training, and it provides the next generation of practitioners with the knowledge and skills needed to provide quality IBD patient care. The Crohn’s & Colitis Foundation plans to continue to provide this vital program in the years to come and will use the feedback from the evaluations and faculty to make curriculum, program, and logistical enhancements.

## Data Availability

Data not publicly available. Day 1:

## Appendix

**Table:**

Adult Curriculum (subject to modifications)
Observe diagnosis and management of the medical aspects of IBD
Observe providers perform history and physical examinations on patients
Observe hospital rounds with IBD NP or GI Consult Faculty
Differentiate between IBD and other types of enterocolitis (infections, ischemia, medication induced, microscopic colitis, celiac, etc.)
Understand indications for and interpretation of diagnostic tests such as colonoscopy, EGD, CT enterography, MR enterography, SBFT, pouchogram, intestinal ultrasound (IUS), pouchoscopy
Appreciate the differences between UC and CD, as well as the subtypes of both diseases
Understand the complications of IBD (obstruction, nutritional, extraintestinal manifestations)
Review the issues of pregnancy and breastfeeding in IBD
Understand the colorectal cancer risk in patients with colonic IBD including surveillance interval, number of biopsies, new methods of managing low-grade dysplasia and polyps
Differentiate between DALM, sporadic adenoma, sessile polyps, pseudopolyps, hyperplastic polyps
Understand psychosocial impact of IBD on well-being and potential methods for improving quality of life
Understand the risks and benefits and dosing of 5-aminosalicylates, immunomodulatory agents, biologics, biosimilars, steroids and antibiotics and their place in therapy, including laboratory monitoring recommendations for immunomodulatory and 5-ASA agents
Review adjunctive medical treatments of bile acid sequestrants, anti-diarrheals, antispasmodics, probiotics, antibiotics, vitamins and nutritional supplements
Understand the indications, potential benefits, risks, contraindications, and complications of appropriate surgical interventions used in the treatment of IBD (ie, ileoanal pouch anastomosis, stricturoplasty, perianal procedures, proctocolectomy with stoma, and diverting stomas)
Understand the recommended follow up, assessment of pouch function, complications and management of patients with an ileoanal pouch (ie, blood work, pouchoscopy with biopsy, and digital exams)
Understand vaccination recommendations and contraindications for patients with IBD
Discuss the relationship between smoking and use of NSAIDS in IBD
Describe the extra-intestinal manifestations that can occur in IBD
Review preventive services that are particularly important in IBD patients (screening for osteoporosis, pap smears)
Understand the nutritional needs (HPN, electrolytes, iron) of patients with short bowel syndrome or chronic malabsorption
Review dietary recommendations for IBD patients with specific nutritional requirements as well as general diet counseling
Review the practice guidelines for UC and CD
Develop treatment recommendations for an IBD case based on presentation (disease severity and extent), patient history, age, gender, support systems available, psychosocial, and nutritional needs
**Pediatric Curriculum (subject to modifications)**
Observe providers perform history and physical examinations on patients
Observe hospital rounds with IBD NP or GI Consult Faculty as available
List types of enterocolitis other than CD or UC and explain the differences between them
List diagnostic tests for IBD and its complications; describe the interpretive criteria
List the differences between UC and CD, as well as the subtypes of both diseases
Choose appropriate medical therapy based on new or existing diagnosis of IBD
List the complications of IBD and when these may occur (obstruction, nutritional, extraintestinal manifestations)
Describe why micronutrient deficiencies occur in IBD; list the labs to be screened to identify these disorders
Name some key elements of managing IBD and school
Discuss growth/nutrition risks and pubertal delay in children with IBD
Describe the psychosocial impact of IBD on well-being and potential methods for improving quality of life
List the risks and benefits and dosing of 5-aminosalicylates, immunomodulatory agents, biologics, steroids and antibiotics and their place in therapy, including laboratory monitoring recommendations for immunomodulatory and 5-ASA agents, identify teratogenic agents
Review adjunctive medical treatments of bile acid sequestrants, anti-diarrheals, antispasmodics, probiotics, antibiotics, vitamins and nutritional supplements
Describe the indications, potential benefits, risks, contraindications and complications of appropriate surgical interventions used in the treatment of IBD, ie, ileoanal pouch anastomosis, stricturoplasty, perianal procedures, proctocolectomy with stoma, diverting stomas, etc., as well as their application and use in pediatrics
Describe the recommended follow up, assessment of pouch function, complications and management of patients with an ileoanal pouch, ie, blood work, pouchoscopy with biopsy, digital exams, etc.
List vaccination recommendations and contraindications for patients with IBD
Discuss the relationship between smoking and use of NSAIDs in IBD
Describe the extraintestinal manifestations that can occur in IBD
Review preventive services that are particularly important in IBD patients (nutritional screening, yearly eye exams, skin exams, and mental health screening)
Read key articles provided and apply knowledge in the clinical setting
Review the nutritional needs (TPN, electrolytes, iron) of patients with short bowel syndrome or chronic malabsorption
Review dietary recommendations for IBD patients with specific nutritional requirements as well as general diet counseling
Review the practice guidelines for UC and CD
Develop treatment recommendations for an IBD case based on presentation (disease severity and extent), patient history, age, gender, support systems available, psychosocial, and nutritional needs
Discuss the use of registries and quality improvement organizations (Improve Care Now)
Discuss the use of enteral therapy/diet therapy in the treatment of pediatric IBD
Identify key components of self-management and transition to adult care
Discuss role of parent/patient partners in care
Discuss role of research in caring for patients with IBD

### 2023 Adult Rotation Schedule Example

#### Day 1:

Introductions with APP Preceptorship Faculty Director

- Goals
- Itinerary/Objectives

IBD Morning Clinic

- Topic discussion—Differences of CD/UC/IBS, diagnostics for workup and health maintenance, pregnancy in IBD, etc.
- Shadow IBD Attending/APP

IBD Afternoon Clinic

- Topic discussions—Medications and monitoring, use of IUS testing in assessing patient, guidelines, protocol discussions, etc.
- Shadow IBD Pharmacist and observe their role in patient management
- Shadow IBD Attending/APP

Wrap-Up: Review the day and check off the objectives/questions covered

#### Day 2:

IBD Morning Clinic: Colon & Rectal Surgery

- Topic discussions—IBD surgical procedures, J-pouch surveillance, post-op management, medical treatments to prevent recurrence. Stoma management and skin issues around stoma, ie, pyoderma gangrenosum, fungal rash, etc.
- Shadow Colorectal APP
- Shadow Stoma/Wound APP
- Virtual or shadow IBD Dietician

IBD Afternoon Clinic: Inpatient APP Patient Rounds with IBD Attending

- Topic discussions—IBD medication management, procedures, complications, extra-intestinal manifestations, discharge planning and follow-up appointments and monitoring, diet instructions and recommendations with IBD dietician
- Virtual or shadow IBD Psychiatrist
- Virtual Patient Support Group Meeting

Wrap-Up: Review the day and check off the objectives/questions covered

#### Day 3:

IBD Morning Clinic

- Topic discussions—IBD new/return visit, medication choices, discuss disease severity, and choice of treatments
- Shadow IBD Attending/APP

IBD Afternoon Clinic

- Topic discussions: Documentation, smart sets, PCP communication letters, case discussion, etc.
- Shadow IBD APP

Wrap-Up: Review the day and final check off to ensure all objectives/questions covered

### 2023 Pediatric Rotation Schedule Example

Introduction with APP Preceptorship Faculty Director

- Introduction to program, discussion of objectives, readings

IBD Morning Clinic with Pediatric Gastroenterologists, APP

Afternoon

IBD Nutrition

- Clinical dietician

IBD Psychology

- Professor of Psychiatry

Pouchitis

- Assistant Professor of Medicine, Division of Gastroenterology Hepatology

Medications

- Assistant Professor of Pediatrics

### Wrap-Up: Review the day and ensure all questions answered

#### Day 2:

Morning

Endoscopy Suite

Afternoon

Health Maintenance

- Associate Professor of Nursing, Division of Gastroenterology

Inpatient and Outpatient Cases

- Professor of Pediatrics, Division Chief of Gastroenterology

IBD Research Studies

- Social/Clinical Research Specialist
- Study Coordinator

Pre-Visit Planning and Nurse Role

- Clinical Nurse, Division of Gastroenterology

Risk Factors for Surgery, Diagnosis of, and Treatment of Common Post-Op Problems in IBD

- Assistant Professor of Surgery

Precision Medicine in Inflammatory IBD

- Professor of Pediatrics, Division of Gastroenterology & Director, Center for Pediatric IBD

ICN Meeting

- All IBD providers

### Wrap-Up: Review the day and ensure all questions answered

#### Day 3:

Morning

Pouch Clinic

- Professor of Medicine & Co-Director, Multidisciplinary Inflammatory Bowel Diseases Center

Ostomy Clinic

- Ostomy nurse

Afternoon

VTE and prophylaxis

- Professor of Pediatrics, Division of Gastroenterology

Outpatient Cases

- Associate Professor of Nursing, Division of Gastroenterology

Testing Results – Interpreting pathology, scopes, and imaging

- Assistant Professor of Pediatrics, Division of Gastroenterology

Case Presentations and Wrap-Up

- Associate Professor of Nursing, Division of Gastroenterology

Wrap-Up: Review the day and final check off to ensure all objectives/questions covered
